# Childhood socioeconomic position and cardiometabolic risk in young adulthood- the impact of mental health

**DOI:** 10.1186/s12889-023-15942-y

**Published:** 2023-06-13

**Authors:** Mia Klinkvort Kempel, Trine Nøhr Winding, Morten Böttcher, Stefan Nygaard Hansen, Johan Hviid Andersen

**Affiliations:** 1 Department of Occupational Medicine, Danish Ramazzini Centre, University Research Clinic, Goedstrup Hospital, Herning, Denmark; 2 Cardiovascular Research Unit, Department of Cardiology, University Research Clinic, Goedstrup Hospital, Herning, Denmark; 3 Department of Public Health, Aarhus University, Aarhus, Denmark; 4 Department of Clinical Medicine, Faculty of Health, Aarhus University, Aarhus, Denmark; 5 Gl. Landevej 61, Herning, 7400 Denmark

**Keywords:** Social inequality, Psychological factors, Cardiometabolic diseases, Young adults, Epidemiology, Causal inference

## Abstract

**Background:**

Low socioeconomic position in childhood is associated with greater cardiometabolic disease risk later in life. The aim of the current study is to examine the mediating impact of mental health on the association between childhood socioeconomic position and cardiometabolic disease risk in young adulthood.

**Methods:**

We used a combination of national registers, longitudinal questionnaire-data and clinical measurements from a sub-sample (N = 259) of a Danish youth cohort. Childhood socioeconomic position was indicated by the educational level of the mother and the father at age 14. Mental health was measured by four different symptom scales at four age-points (age 15, 18, 21 and 28), and combined into one global score. Cardiometabolic disease risk was measured by nine biomarkers at age 28–30 and combined into one global score by sample-specific z-scores. We conducted analyses within the causal inference framework and evaluated the associations using nested counterfactuals.

**Results:**

We found an inverse association between childhood socioeconomic position and cardiometabolic disease risk in young adulthood. The proportion of the association which was mediated by mental health was 10 (95% CI: -4; 24) % and 12 (95% CI: -4; 28) % using educational level of the mother and the father as indicator, respectively.

**Conclusions:**

Accumulated poorer mental health in childhood, youth and early adulthood partially explained the association between low childhood socioeconomic position and increased cardiometabolic disease risk in young adulthood. The results of the causal inference analyses rely on the underlying assumptions and correct depiction of the DAG. Since these are not all testable, we cannot exclude violations that potentially could bias the estimates. If the findings can be replicated, this would support a causal relationship and direct potentials for intervention. However, the findings point to a potential for intervention in young age in order to impede the translation of childhood social stratification into later cardiometabolic disease risk disparities.

**Supplementary Information:**

The online version contains supplementary material available at 10.1186/s12889-023-15942-y.

## Background

There is a substantial social disparity in the prevalence of cardiometabolic diseases in most countries [1, 2]. Not only adult socioeconomic position (SEP) but also childhood SEP is associated with cardiometabolic disease risk in adulthood [3, 4]. Multiple studies examine the association between childhood SEP and later cardiometabolic disease risk. However, the pathways through which differences in social stratification “get under the skin” and translate into disparities in cardiometabolic disease risk is not entirely clear [5]. Some of the association between childhood SEP and later cardiometabolic disease risk can be explained by differences in lifestyle and potential differences in the vulnerability to unhealthy lifestyle factors across SEP [6]. Another possible explanation is the effect of mental health. Observational studies show that children growing up in families with lower SEP experience poorer mental health as compared to children growing up in families with higher SEP [7]. This include higher levels of perceived stress, depressive symptoms and a lower degree of self-esteem and coherence, which have been linked to increased cardiometabolic disease risk [8–10]. Poor mental health is suggested to cause undesirable physiological effects in multiple biological domains related to cardiometabolic disease risk, e.g. inflammation, lipids, blood pressure and glucose-metabolism and has become a recognised risk factor for cardiometabolic diseases [11, 12]. Altogether, this proposes a mediating role of mental health in the association between childhood SEP and later cardiometabolic disease risk.

Different studies use different biomarkers and a variety of composite measures to assess cardiometabolic disease risk in association with social circumstances [13]. The development of composite measures is consistent with growing evidence that pathological effects operate through an interplay of different physiological domains in an additive or synergistic manner. Common to all of the composite measures are the inclusion of multiple (patho)physiological domains often reflecting the cardiovascular system, metabolic system, inflammatory system and neuroendocrine system [13, 14]. However, the included biomarkers and the construction of the different composite measures vary between studies [13–15].

Improved understanding of the pathways and potentials to intervene upon the effects of lower childhood SEP on later cardiometabolic disease risk is critical to inform policy makers and improve prevention strategies to combat the increasing social disparity in cardiometabolic disease risk. In the current study, we hypothesize that accumulated poor mental health *(*depressive symptoms, low sense of coherence, high perceived stress and poor self-esteem combined into one global score*)* in childhood, youth and early adulthood (ages 15–28) mediates some of the association between childhood SEP and overall cardiometabolic disease risk at age 28–30.

## Methods

### Study population

**Fig. 1:**
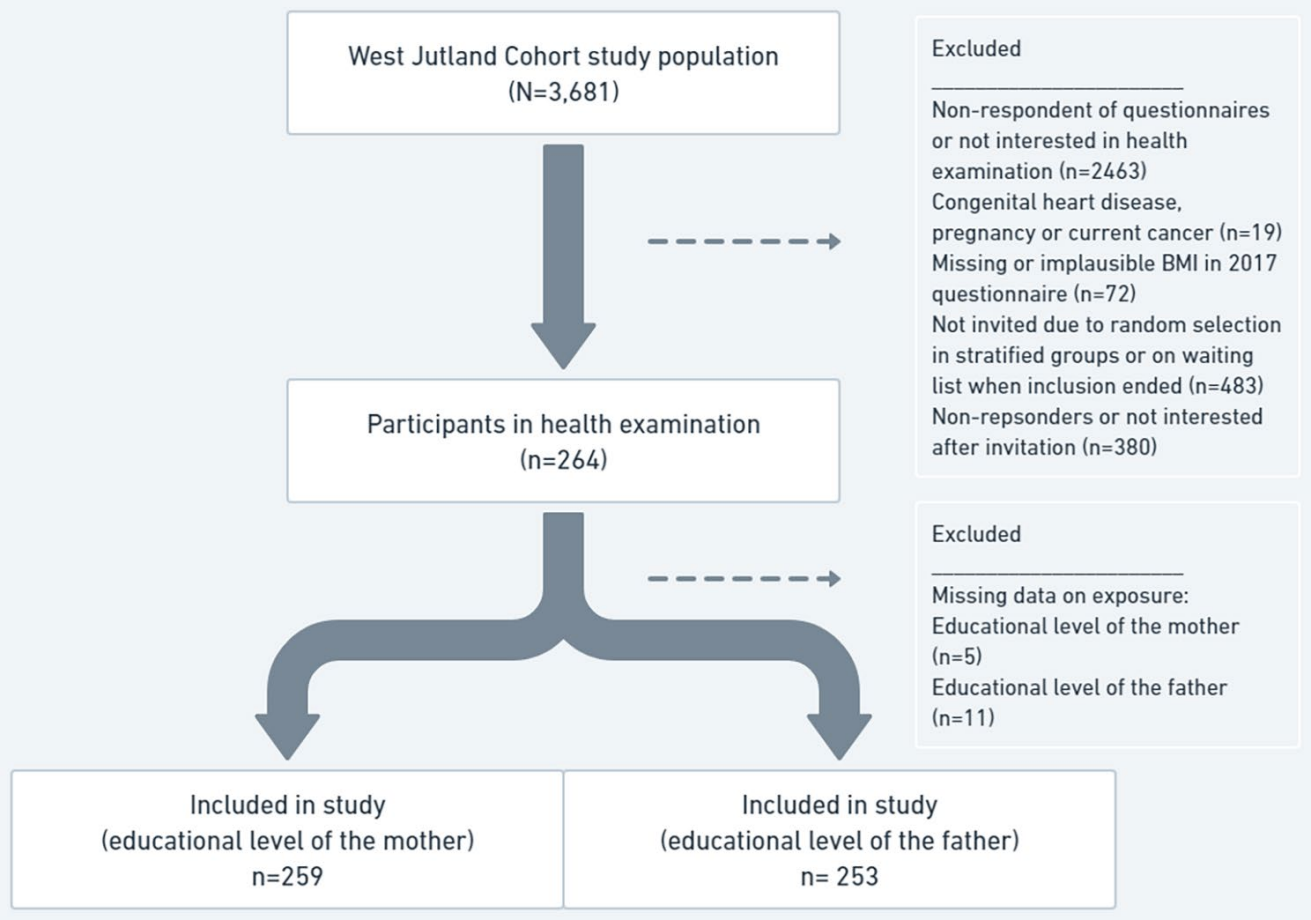
Flowchart of the study population

This study included a sub-sample (n = 259) of participants from the ongoing West Jutland Cohort Study [16]. The cohort study comprised all individuals born in 1989 and living in a specific county in Western Denmark in 2004 (N = 3,681) [17]. All participants were invited to fill in questionnaires regarding lifestyle, physical health and psychosocial factors at ages 15, 18, 21 and 28. Study participants were invited into the current study if they had responded to the initial and latest questionnaires and had indicated interest in a health examination. Invitations to be included in the health examination were based on sex and self-reported body mass index (BMI) (< 25, 25–30, > 30 kg/m2) at age 28 as described in detail previously [16]. A flowchart illustrating the study population is presented in Fig. 1.

### Exposure

Various indicators with various meanings and interpretations are used to measure SEP in relation to health at different ages [18]. Based on prior studies from the same study population we decided to use parental educational level as indicator of childhood SEP [19, 20]. This indicator reflects psychosocial aspects of SEP rather than material aspects and appears to be the best indicator of childhood SEP in a Danish welfare society [18, 19]. The educational levels of the mother and father were investigated separately. Data included highest level of parental education when the participant was 14 years. Data was derived from educational registers obtainable from Statistics Denmark and dichotomized based on years of completed education into low (≤ 10 years), which in Denmark is equivalent to compulsory school, and high (> 10 years) [21].

### Outcome

All biological measures were obtained from a health examination when the participants were 28–30 years [16]. The health examination was conducted by trained nurses using standardized operating procedures which are described in detail previously [16]. Based on prior work, we created a continuous cardiometabolic risk (CMR) score from 9 biomarkers covering four different biological domains (inflammation, lipids, glucose-metabolism and blood pressure) in order to capture the overall cardiometabolic disease risk in young adulthood [15, 22]. The biomarkers included in each domain are presented in supplementary Table S.1. All biomarkers were standardized for each sex separately and inverse probability weights were applied to account for the sampling by BMI. We created a continuous score by summarizing the mean values of the four domains. This overall score was then subsequently standardized. Higher scores indicate higher cardiometabolic disease risk.

### Mediator

This study investigated the mediating impact of accumulated mental health (hereafter mental health) across childhood, youth and early adulthood rather than a single measure at a single age-point. To evaluate the overall and accumulated influence of mental health, we created a continuous, composite mental risk score by standardizing and summarizing four established symptom scales of the entire West Jutland Cohort Study population across four age-points. The psychological factors included in the score have previously been associated with SEP and cardiometabolic disease risk and comprise; sense of coherence, self-esteem, depressive symptoms and perceived stress [10]. The first two factors are inversely related to the latter two and thus multiplied by -1 prior to summarizing them. Prior to this we conducted a factor-analysis and displayed the variation as a scree plot to support the use of one global score. All psychological factors were measured based on data from questionnaires at ages 15, 18, 21 and 28. Participants were included in the study if they had responded to at least two psychological measures at least two times. Higher scores indicated poorer mental health. The specific response rates concerning the psychological measures of the study participants and the overall cohort population are presented in supplementary Table S.2 and S.3.

Sense of coherence is believed to capture the ability of an individual to understand, manage and make sense of various life-situations in order to cope efficiently. In the current study we used a measure of meaningfulness evaluated by a revised 4-item short version of the original 29-item questionnaire proposed by Antonovsky [23]. Self-esteem is defined as the individual’s “*attitude of approval or disapproval toward oneself”* and was measured by a 6-item short version of the original 10-item scale by Rosenberg [24]. Depressive symptoms were measured by a 4-item short version of the Center for Epidemiological Studies Depression Scale for children, adolescents and young adults (CES-DC) [25]. The scale is a general measure of psychopathology rather than a measure of depressive disorder. Perceived stress is the subjective measure of appraised stress by the individual. In the current study we used a Danish 4-item version of the original 14-item Perceived Stress Scale by Cohen at ages 15, 18 and 21, and a 10-item version at age 28 [26]. The specific items used in the questionnaires of the current study are presented in supplementary Table S.4.

### Causal structures

**Fig. 2:**
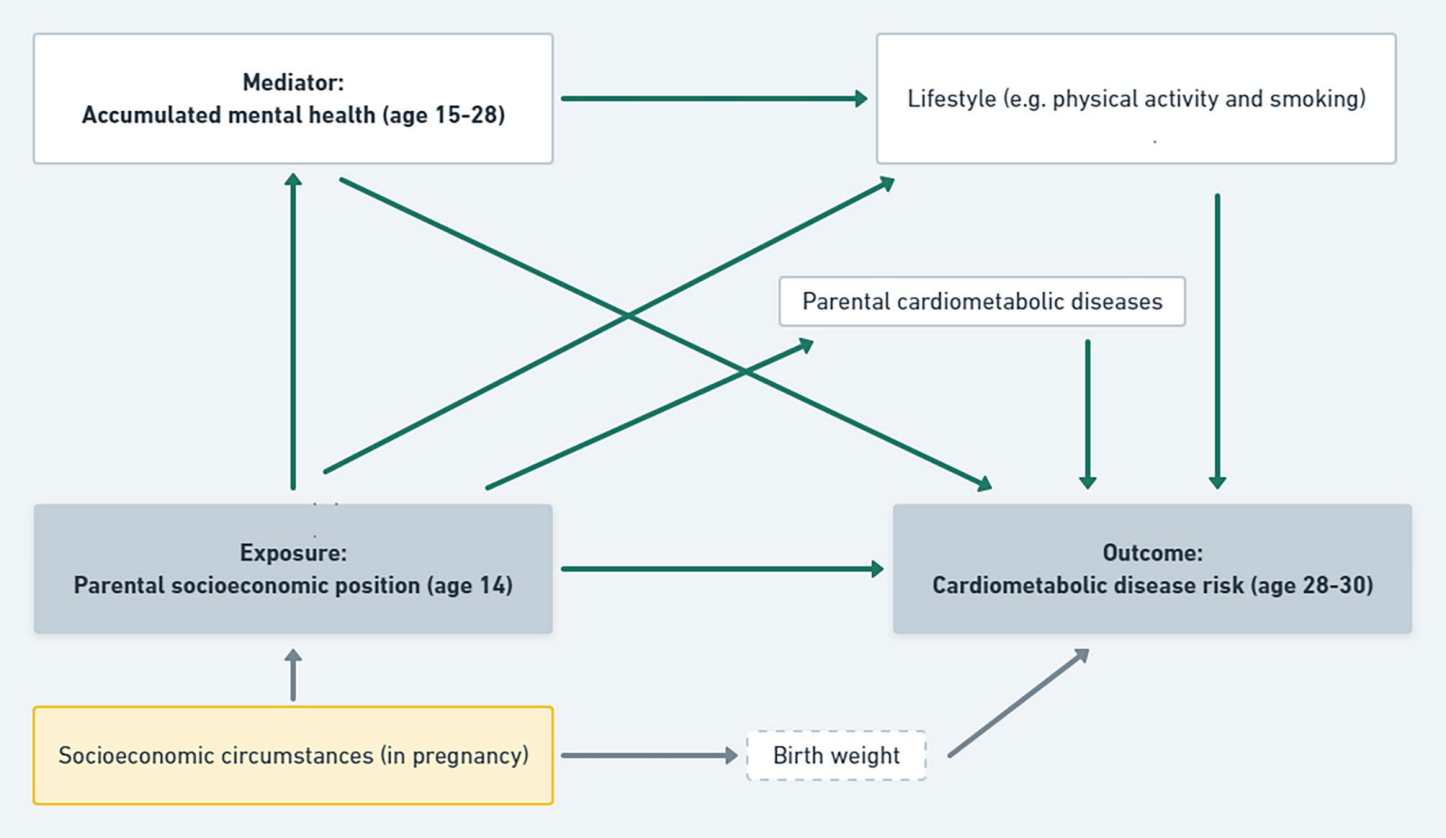
Simplified Directed acyclic graph of assumed structures Legend: Grey boxes refer to exposure and outcome, white boxes refer to mediators, dotted white box refers to back-door that are included as confounder, thus the path is “closed”

The study was conducted within the causal inference framework and analyses were guided by a directed acyclic graph (DAG) constructed based on knowledge about the assumed structures from the existing literature. In order to provide transparency of the causal structures used in the study a simplified DAG is presented below in Fig. 2. Nodes/arrows not relevant for the final model or described in the manuscript were not included in the simplified DAG. Using a DAG ensures identification of relevant confounders without introducing unwanted bias, e.g. collider bias, assuming that the DAG sufficiently depicts the causal structures. Moreover, the DAG captures the overall associations and makes sure not to control for intermediate variables on the path of interest and thus underestimate the effect by decomposing it into more parts. Below we will present some of the considerations underlying the nodes and arrows in the DAG. A particular focus will be on some of the usual confounders which are not included in the current study. Furthermore, details about response rates and the components of the mediator and outcome variables are shown in supplementary Table S.1-S.4.

### To be or not to be a confounder

Below we will summarize some of the considerations underlying the depiction of the structures presented in the DAG above.

Birth weight: As indicated in the DAG above (Fig. 2), there is a backdoor through birth weight on the exposure-outcome path. Several studies find associations between low birth weight, acting as an indicator of poor conditions in utero, and increased cardiometabolic disease risk [27]. Furthermore, epidemiological studies show that new-borns from lower SEP families have lower birth weight as compared to new-borns from higher SEP families [28]. Based on the DAG above it is thus necessary to include birth weight as a confounder for the exposure-outcome relationship. Information about birth weight was derived from the Danish Medical Birth Register obtained at Statistics Denmark. However, due to approximately 4% missing values in the register we supplemented the data with data from parental questionnaires when the child was 15 years old. Birth weight was used as a continuous measure.

Physical activity: Level of physical activity is related to cardiometabolic disease risk, childhood SEP and mental health. Individuals with better mental health are more likely to be physically active as compared to those with poorer mental health [29]. However, most studies are cross-sectional and the direction and temporal nature of the relationship are thus difficult to determine [30, 31]. Recently, Martins et al. published an updated systematic review of qualitative studies concerning adolescent perspectives on barriers and facilitators of physical activity [32]. The authors describe individual (e.g. psychological factors) and social/relational factors as two of the major areas for barriers/facilitators for physical activity. Based on this knowledge we chose to grasp mental health as an antecedent of the level of physical activity. However, due to the uncertainty in the literature as to the direction of the association, we further conducted supplementary analyses including physical activity as a confounder of the mediator-outcome relationship. This analysis is presented in supplementary Figure S.1. Information about physical activity was derived from questionnaires at ages 15, 18, 21 and 28.

Parental cardiometabolic diseases: In addition to its association with SEP, cardiometabolic disease inheritance across generations are well established [33]. However, manifest cardiometabolic diseases primarily occur in older age with a peak around age 55–74 for men and 65–84 for women [34]. This means that parental cardiometabolic diseases primarily establish after parental attainment of educational level (exposure). Also, parental cardiometabolic diseases are not considered to affect mental health of their children in childhood and young adulthood (mediator) and are thus not included in the current study as a confounder.

Sex: Sex-differences in mental health (mediator) and cardiometabolic disease risk in early adulthood (outcome) are evident [35]. To account for this the mediator and outcome variables were standardized sex-stratified as described above. We further acknowledge the literature pointing towards potential moderating effects of sex in the exposure-mediator and exposure-outcome paths. Consequently, we inserted an interaction term between sex and SEP in all analyses.

### Statistical analyses

All data were analysed with Stata software version 16.1 (Stata corporation, College Station, Texas). Initially, descriptive statistical analyses were conducted to present demographics, mental risk score and cardiometabolic risk score by exposure level. We used the counterfactual notation and G-computation to facilitate the possibility of comparing mean CMR score values under different scenarios as described in detail elsewhere [36]. Since, in our DAG, the outcome (Y) is affected by the exposure (X) directly and through the mediator (M), the counterfactual outcome is a nested counterfactual Y(x,M(z)), which corresponds to the value of the outcome had the individual been exposed to x and had the mediator been set to its value, M(z) under exposure z. The average total effect of SEP (exposure) on CMR (outcome) can be defined as the hypothetical contrast had everyone been exposed versus had no one been exposed. In this notation, the contrast corresponds to E(Y(1,M(1)))-E(Y(0,M(0))). The average total effect may then be decomposed into the pure direct effect, PDE = E(Y(1,M(0)))-E(Y(0,M(0))), and the total indirect effect, TIE = E(Y(1,M(1)))-E(Y(1,M(0))). The proportion mediated (PM) through mental health is thus defined as PM = TIE/(TIE + PDE) with TIE + PDE being the average total effect of SEP on CMR. The PM may be negative when TIE and TIE + PDE are in opposite directions, or positive when TIE and TIE + PDE are in the same direction. We fitted the models with variables as described above and further applied inverse probability weights in all analyses to account for the sampling by sex and BMI. We applied bootstrapping with 100 replications to obtain valid confidence intervals.

## Results

As depicted in Table 1, a total of 259 participants were included in the study. Individuals from families with lower SEP had increased mean levels of mental and cardiometabolic risk scores as compared to individuals from families with higher SEP. This is evident for the global scores of both mental risk and cardiometabolic risk and for all distinct domains included in the two risk scores.

**Table 1:** Descriptive statistics of socioeconomic position, mental health and cardiometabolic risk score by parental educational level

	Educational level of the mother N = 259		Educational level of the father N = 253	
	Low	High	Low	High
Total, n (%)	63 (24.3)	196 (75.7)	74 (29.2)	179 (70.8)
Men	28 (21.5)	102 (78.5)	37 (29.1)	90 (70.9)
Women	35 (27.1)	94 (72.9)	37 (29.4)	89 (70.6)
Own Educational level, n (%)				
> 13 years	31 (19.3)	130 (80.7)	36 (22.9)	121 (77.1)
11–13 years	25 (32.5)	52 (67.5)	25 (33.3)	50 (66.7)
≤ 10 years	7 (33.3)	14 (66.7)	13 (61.9)	8 (38.1)
Mental risk score *, mean (SD)	0.06 (0.52)	-0.03 (0.60)	0.06 (0.57)	-0.03 (0.59)
CES-DC *	0.06 (0.65)	-0.02 (0.71)	0.08 (0.76)	-0.02 (0.69)
Perceived Stress *	0.00 (0.68)	-0.08 (0.68)	0.04 (0.69)	-0.08 (0.67)
Sense of Coherence, inverse *	0.01 (0.56)	-0.01 (0.72)	0.05 (0.66)	-0.02 (0.67)
Self-esteem, inverse *	0.18 (0.71)	-0.03 (0.75)	0.07 (0.70)	0.00 (0.76)
Cardiometabolic risk score *, mean (SD)	0.51 (0.94)	0.12 (1.04)	0.52 (0.98)	0.11 (1.08)
Inflammation *	0.64 (1.04)	0.08 (0.96)	0.42 (0.97)	0.15 (1.04)
Lipid status *	0.37 (1.02)	0.07 (0.99)	0.40 (1.01)	0.06 (1.01)
Glucose metabolism *	0.25 (0.88)	0.13 (1.15)	0.37 (1.16)	0.10 (1.11)
Blood pressure *	0.16 (0.76)	0.04 (1.04)	0.25 (1.07)	-0.01 (0.94)

Data are presented as mean (SD) for continuous measures, and n (%) for categorical measures. * Standardized values, with sample-weights applied. CES-DC, Center for Epidemiological Studies Depression Scale for children.

**Fig. 3:**
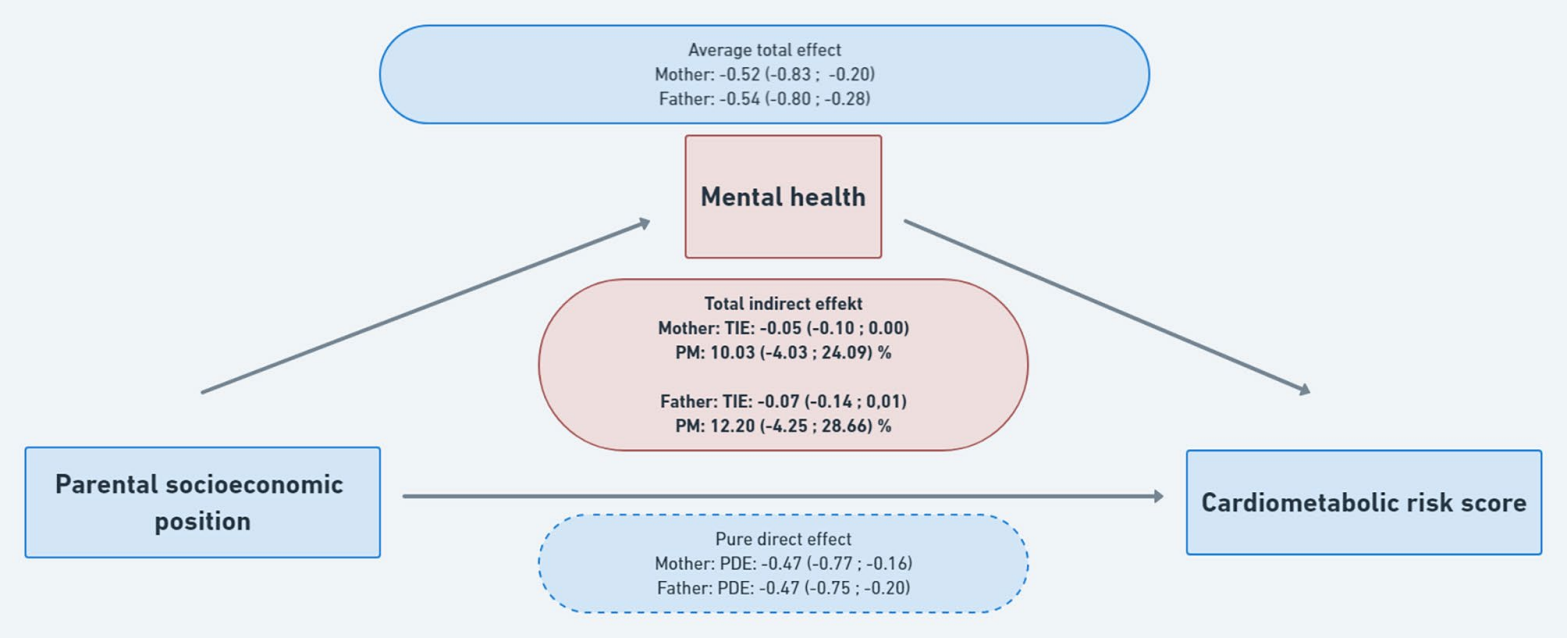
The mediating impact of mental health on the association between childhood socioeconomic position and cardiometabolic risk score Legend: TIE, total indirect effect. PM, proportion mediated. PDE, pure direct effect. All estimates are presented with 95% confidence intervals

The results from the G-computation analyses are presented in Fig. 3. The average total effects of childhood SEP on the cardiometabolic risk score are − 0.52 (95% CI: -0.83; -0.20) and − 0.54 (95% CI: -0.80; -0.28) using educational level of the mother and father as indicator, respectively. This corresponds to the hypothetical contrast of an increased cardiometabolic risk score if everyone was growing up in families with low SEP versus everyone was growing up in families with high SEP. Decomposing these estimates, the pure direct effects, which are explained by other factors than mental health, are similar for the two indicator variables: -0.47 (-0.77; -0.16) and − 0.47 (-0.75; -0.20). The total indirect effects, explained by mental health, are − 0.05 (-0.10;0.00) and − 0.07 (-0.14;0.01), using educational level of the mother and the father, respectively. The proportion of the increased cardiometabolic risk score mediated by mental health is thus 10.03 (95% CI: -4.03; 24.09) % and 12.20 (95% CI: -4.25; 28.66) %, respectively.

## Discussion

In this study we examined the mediating impact of accumulated mental health in childhood, youth and early adulthood on the association between childhood SEP, indicated by parental educational level, and cardiometabolic disease risk at age 28–30. To investigate the association, we used G-computation and nested counterfactuals on longitudinal data. The primary results were that childhood SEP was inversely associated with cardiometabolic disease risk and that 10–12% of the association could be mediated through accumulated mental health. However, the estimates of the proportion mediated were fairly uncertain with confidence intervals ranging from an inverse effect of mental health (-4%) to 29%.

A variety of elements might explain the robust association between childhood SEP and later cardiometabolic disease risk. Some of those are adverse health behaviours, less extracurricular activities, poor housing quality and environmental pollutants [37]. These are all elements that accumulate in lower SEP environments. Recent literature further suggests psychosocial factors as mediators of the association [15, 38]. However, empirical studies show mixed results. Doom et al. examined mediators between adolescent SEP and cardiovascular disease risk in young adulthood, mean age 28.9 years, by path analyses on data from the National Longitudinal Study of Adolescent to Adult Health (N = 14,493) [22]. They found indirect paths through health behaviour and educational attainment but not through depressive symptoms. Some methodological aspects might challenge these findings. For instance, all mediators were measured simultaneously at one age-point and potential causal relationships between the mediators were not included, e.g. earlier depressive symptoms might be an ancestor of both educational attainment and health behaviour as previously described. Furthermore, the outcome measure was a Framingham based composite score including smoking status, which might be related to mental health through maladaptive coping mechanisms. Additionally, statistical challenges when analysing multiple mediators by path analyses might hinder firm conclusions about causality [39]. In a study from the 1958 British Birth cohort (N = 6,027) Winning et al. found that 37% of the association between childhood social disadvantage and cardiometabolic risk at age 45 was mediated through childhood distress [15]. These results are in line with our results however, the magnitude of the proportion mediated is larger. This might partly be explained by differences in the exposure variables. Winning et al. created an index from 16 exposures related to family and socioeconomic hardship. This might strengthen the association with psychological distress and thus increase the proportion mediated.

There is no consensus about the best measure of mental health when examining the impact on the association between SEP and health outcomes [38]. Recent research suggests an integrative approach concerning measures of mental health and recommends the use of composite scores to capture the overall and shared biological and behavioural effects [40]. However, Ryff et al. recommend separate measures of mental well-being and mental ill-being as they might describe different aspects of mental health [41]. To comply with this, we conducted supplementary analyses (not shown) for each psychological measure individually and for combinations of mental well-being (self-esteem and sense of coherence) and ill-being (perceived stress and depressive symptoms). All results were attenuated compared to using the global score which could indicate an increased robustness of the global score as compared to distinct measures in order to capture the overall mediating effect in the association. Conducting G-computation with a continuous mediator and outcome, VanderWeele et al. showed that non-differential measurement error of the mediator will bias the indirect effect towards the null and the direct effect away from the null [42]. Adding further psychological measures to the global score could potentially strengthen this measure even more thus increasing the proportion mediated. The composite approach facilitates fusion of complex structures into overall mental health status. However, this approach requires complementary research in order to differentiate potential mediating effects of specific timing and specific mental health measures in the association. This remains important for future research.

Various mechanisms can plausibly explain the potential impact of mental health on the relationship between childhood SEP and cardiometabolic disease risk. Some of these are direct neurobiological modulations in childhood which can impact later health negatively through different pathophysiological mechanisms. This includes structural changes in grey and white matter and altered metabolism of neurotransmitters involving serotonin, dopamine and glutamate [43]. One of the other mechanisms relate to expected “acute” responses from the hypothalamus-pituitary-adrenal axis that can have prolonged impact when the negative feedback system is impaired due to persistent activation [44]. Furthermore, acute and chronic psychosocial stress alter the autonomic nervous system with sympathetic predominance which further influences multiple biological domains related to cardiometabolic disease risk [45].

### Strengths and limitations

Some notable strengths of the present study include multiple measures of mental health from multiple age-points, register based information on SEP and a robust measure of cardiometabolic disease risk respecting the potential additive/synergistic effect of various biological domains. We cannot exclude that the findings are affected by reverse causation. However, the longitudinal study design with register based information on parental educational level, self-reported measures of mental health at ages 15–28 (2004–2017) and objectively measured cardiometabolic risk at age 28–30 (2018–2019) prior to manifest disease give some reassurance that the findings are not simply due to reverse causation. Also the use of different data sources decreases the risk of common method bias. In addition, both mediator and outcome were continuous measures instead of arbitrary cut-off values. Finally, using G-computation rather than traditional statistical approaches towards mediation analyses was seen as a strength in the current study context [46, 47]. However, the results of the causal inference analyses rely on the underlying assumptions and correct depiction of the DAG. This is discussed in the methods section and will be further discussed below.

Our study has some limitations that need to be addressed. Most importantly, the study is based on a sub-sample of a cohort study and non-respondents and attrition might bias the results. Additional analyses, stratified by self-reported BMI- and sex, showed that overall questionnaire-responders have poorer mental health compared to the study-participants (supplementary Table S.5). In general, mental health is inversely related to BMI. However, men with BMI > 30 kg/m^2^ participating in the study have better mental health than men with BMI 25–30 kg/m^2^. Since BMI is further associated with cardiometabolic disease risk this selection might bias the total indirect effect towards the null. However, running the G-computations without men with BMI > 30 kg/m^2^ did not change the estimates noticeably. A former study investigating the initial non-participation in the West Jutland Cohort study finds that individuals from lower SEP are more likely to be non-responders [17]. If non-responders also have increased cardiometabolic disease risk this might have underestimated the total effect and thus overestimated the total indirect effect. However, if non-responders in addition have poorer mental health this might have underestimated the total indirect effect thus pointing the proportion mediated towards the results of the current study. The study population was sampled within strata of body mass index and sex thus increasing the heterogeneity of the study-population. Afterwards we applied inverse probability weights. This re-weighting is intended to generate a pseudo-population that, on average, mimics the background population. However, it is important to recognize that the weights are only valid if the respondents and participants truly represent the non-respondents and non-participants of each stratum of factors used to generate the probabilities. This is potentially not the case, and we cannot exclude that some selection bias or sample selection bias remain. Furthermore, the sample size could be a limitation that might explain the rather wide confidence intervals of the estimates. As mentioned earlier, there is no consensus about the best measure of mental health. Since subclinical measures of mental health are subjective in nature, it seems reasonable to evaluate by self-reporting. However, some concerns need to be addressed. Firstly, there is a risk of interpretation difficulties and over- or underreporting of each symptom scale by the respondent. Secondly, measures of mental health may possibly vary over time and consequently depend on time of measurement. Thirdly, the use of abbreviated scales could result in loss of information and thus inaccurate measures. All of the abovementioned concerns can lead to misclassification that we expect would be non-differential. We created the accumulated composite score from different measures of mental health across different age points. This was done in an attempt to overcome some of the concerns mentioned above, to induce robustness to the measure and capture the overall mental health of the participant. However, despite the effort to create a more robust measure of mental health, some non-differential measurement error most likely remains, and we cannot exclude that the proportion mediated in our study is underestimated. The average total effect is defined as the hypothetical contrast had everyone been exposed versus had no one been exposed. As this information in its nature cannot be acquired, we have to rely on three core assumptions to justify the analyses: counterfactual consistency, conditional exchangeability and positivity [48–51]. *Counterfactual consistency* requires that the potential outcome given the actual exposure is equal to the observed outcome. This assumption generally holds when the exposure is well defined. The use of causal inference in social epidemiology has been the subject of debate with a particular focus on the consistency assumption [46, 47, 52]. Galea and Hernán argue that causal inference should be a key goal for social epidemiology and further highlight that experimental manipulation is not a requirement for meaningful causal inference [46]. Regarding the current study, we find it reasonable to assume consistency using educational level as an indicator of SEP. Especially in a Danish setting with free and equal access to education and a rather high degree of consistency across schools regarding e.g. school duration and quality. However, since this is not testable, we cannot fully exclude violation of this assumption. *Conditional exchangeability* is analog to no uncontrolled or residual confounding and no selection bias. We used a DAG to identify potential confounders. In a DAG, confounders are identified through the back door criterion assuming that the DAG sufficiently depicts the causal structures [53]. A potential violation of the assumption of no uncontrolled confounding in the relationship between exposure and mediator could be early manifest parental cardiometabolic disease or parental mental health. This is the case if parental mental health is a descendent of previous mental health which has affected parental educational attainment. Furthermore, parental mental health may affect the mental health of the offspring. Supplementary analyses using parental self-reported psychiatric disorders in 2004 as a proxy for mental health changed the proportion mediated to 13% and 16% using the educational level of the mother and father, respectively. This induces some assurance regarding the original findings. However, we cannot exclude that some residual confounding remains. This could be caused by misclassification of the observed confounders or unmeasured confounders that we were not aware of. Furthermore, the study population of the West Jutland Cohort is restricted to individuals born in 1989. Thus, age as a potential confounder was eliminated by design. Selection bias was discussed above. In addition to this, the use of inverse probability weights reduces the risk of selection bias from inclusion by sex and BMI. We did supplementary analyses to account for non-respondents of the questionnaire in 2017. We calculated the supplementary weights based on educational level of the mother and sex, which was available from registers. In order to ensure stability of the weights we did not include more variables. Adding these weights did not change the proportion mediated noticeably (PM = 11% and 12% evaluating the educational level of the mother and the father respectively). *Positivity* refers to the probability of being either exposed or unexposed, which has to be non-zero for every combination of exposures and confounders. In the current study, both unexposed and exposed individuals are included in categories of sex and birthweight. Under these assumptions, we can justify the use of G-computation on nested counterfactuals.

### Conclusion

Our study finds that decreased mental health in childhood, youth and early adulthood in part explains the association between lower childhood SEP, indicated by parental educational attainment, and later cardiometabolic disease risk. Based on our findings, we argue that intervention should not only focus on traditional risk factors, such as diet, smoking and physical activity, but also on a potential ancestor of these factors, mental health, with highly social stratification. This could preferably be carried out across various sectors in society in a multidisciplinary approach including not only the healthcare sector but also other parts of society such as day care facilities and schools. While increasing numbers of studies examine the association between various measures of childhood SEP and cardiometabolic disease risk, specific knowledge about the underlying mechanisms is incomplete. The responsibility of the research community includes uncovering and quantifying potential targets for intervention in the association between low childhood SEP and later disease risk. Despite the risk of bias in epidemiological studies, we believe these are essential to inform policy makers and improve upstream cardiometabolic prevention strategies. The findings of the current study should preferably be supplemented with other longitudinal studies in larger population samples in different settings, including sex-stratified analyses. If the findings can be replicated, this would support a causal relationship and direct potentials for intervention. In addition to this, studies examining other mediators in the relationship would be of great interest in order to further quantify and disentangle how childhood SEP “gets under the skin”.

## Electronic supplementary material

Below is the link to the electronic supplementary material.

Supplementary Material 1

## Data Availability

Due to confidentiality restrictions apply to the availability of the data analysed during this study. The corresponding author will on request detail the restrictions and any conditions under which access to some data may be provided.
