# Supplementary material for: Childhood socioeconomic position and cardiometabolic risk in young adulthood- the impact of mental health
Source: BMC Public Health. 2023 Jun 13;23:1136. doi: 10.1186/s12889-023-15942-y (PMC10265760; doi:10.1186/s12889-023-15942-y)
Supplement: Supplementary file 1 — Supplementary Material 1 [file 12889_2023_15942_MOESM1_ESM.pdf]

Supplementary:

*Table S.1. Biomarkers included in the four biological domains of the outcome*

| Biological domain | ***Biomarkers*** |
|---|---|
| Inflammation | High-sensitive CRP |
| | Interleukin-6 |
| | Fibrinogen |
| Lipid status | Inverse High-density lipoprotein cholesterol |
| | Triglycerides |
| Glucose metabolism | Insulin |
| | Glucose |
| Blood pressure | Systolic blood pressure |
| | Diastolic blood pressure |

*Table S.2. Number of study participants in health examination (N=264) who responded to each psychological variable in each wave*

| **Psychological variable, n (%)** | **Wave 1** ***15* years** | **Wave 2** ***18*  years** | **Wave 3** ***21 years*** | **Wave 4** ***28 years*** |
|---|---|---|---|---|
| **Depressive symptoms** | 263 (99.6) | 221 (83.7) | 205 (77.7) | 261 (98.9) |
| **Sense of coherence** | 262 (99.2) | 220 (83.3) | 207 (78.4) | 261 (98.9) |
| **Perceived stress** | 262 (99.2) | 219 (83.0) | 210 (79.5) | 264 (100.0) |
| **Self-esteem** | 261 (98.9) | 221 (83.7) | 209 (79.2) | 261 (98.9) |

*Table S.3 Number of participants in the overall cohort (N=3,681) who responded to the psychological variables in each wave*

| **Number of responses to the four psychological variables, n (%)** | **Wave 1** ***15*** **years** | **Wave 2** ***18*** **years** | **Wave 3** ***21 years*** | **Wave 4** ***28 years*** |
|---|---|---|---|---|
| **0** | 628 (17.1) | 1,286 (34.9) | 1,680 (45.6) | 1,714 (46.6) |
| **1** | 6 (0.2) | 11 (0.3) | 5 (0.1) | 61 (1.7) |
| **2** | 30 (0.8) | 9 (0.2) | 27 (0.7) | 12 (0.3) |
| **3** | 139 (3.8) | 98 (2.7) | 111 (3.0) | 74 (2.0) |
| **4** | 2,878 (78.2) | 2,277 (61.9) | 1,858 (50.5) | 1,820 (49.4) |

*Table S.4. Items included in the psychological variables*

| Mental health variable | Questionnaire items, age 15, 18, 21 and 28 |
|---|---|
| Depressive symptoms | "During the past week, how much have you had the following feelings" (response categories: "not at all", "a little", "some" and "a lot")<br>a) "I was happy this week"<br>b) "I felt like kids I knew were not friendly or that they didn't want to be with me" (at age 28: "I felt people were not friendly")<br>c) "I felt sad"<br>d) "It was hard to get started doing things this week" |
| Sense of coherence | 1. How do you feel about the things you do every day?<br>(response categories: "very interesting", "interesting", "OK", "boring" and "very boring")<br>2. About your daily life:<br>(response categories: "very often", "often", "sometimes, "almost never" and "never")<br>a) "How often do you do things that you think are meaningful?"<br>b) "How often do you have the feeling that you don’t really care about what goes on around you?"<br>c) "How often do you have the feeling that there is little meaning in the things you do?" |
| Perceived stress | "In the last month, how often...?"<br>(response categories: "very often", "fairly often", "sometimes", "almost never" and "never") |

| | |
|---|---|
| | a) "have you felt that you were unable to control the important things in your life?"<br>b) "have you felt confident about your ability to handle your personal problems?"<br>c) "have you felt that things were going your way?"<br>d) "have you felt difficulties were piling up so high that you could not overcome them?"<br>- At age 28 furthermore:<br>e) "have you been upset because of something that happened unexpectedly?"<br>f) "have you felt nervous and stressed?"<br>g) "have you found that you could not cope with all the things that you had to do?"<br>h) "have you been able to control irritations in your life?"<br>i) "have you felt that you were on top of things?"<br>j) "have you been angered because of things that were outside your control?" |
| Self-esteem | "How much do you agree or disagree with the following statements? There are no right or wrong answers"<br>(response categories: "strongly agree", "agree", "disagree", and "strongly disagree")<br>a) "I feel that I have a number of good qualities"<br>b) "I feel that I'm a person of worth at least equal to others"<br>c) "I am able to do things as well as most other people" |

| | |
|---|---|
| | d) "I take a positive attitude toward myself"<br><br>e) "On the whole, I am satisfied with myself"<br><br>f) "All in all, I'm inclined to feel that I'm a failure" |

Legend: All questions and response categories were in Danish. The original questionnaires are available in Danish at www.vestliv.dk

*Table S.5. Mental risk score by self-reported Body Mass Index (BMI) at age 28*

| | N | BMI<25 kg/m² | BMI 25-30 kg/m² | BMI>30 kg/m² |
|---|---|---|---|---|
| Participants | **264** | -0.16 (0.50) | 0.02 (0.55) | 0.14 (0.69) |
| Men | 132 | -0.16 (0.56 | 0.02 (0.54) | -0.03 (0.68) |
| Women | 132 | -0.16 (0.42) | 0.02 (0.58) | 0.28 (0.67) |
| All respondents | **1819** | -0.08 (0.58) | 0.03 (0.62) | 0.16 (0.68) |
| Men | 753 | -0.07 (0.60) | 0.01 (0.59 | 0.06 (0.69) |
| Women | 1066 | -0.08 (0.57) | 0.06 (0.65) | 0.23 (0.67) |

Data are presented as mean (SD). Higher score indicates poorer mental health.

*Supplementary Figure S. 1 Results of supplementary analyses when further adjusting for level of physical activity.*

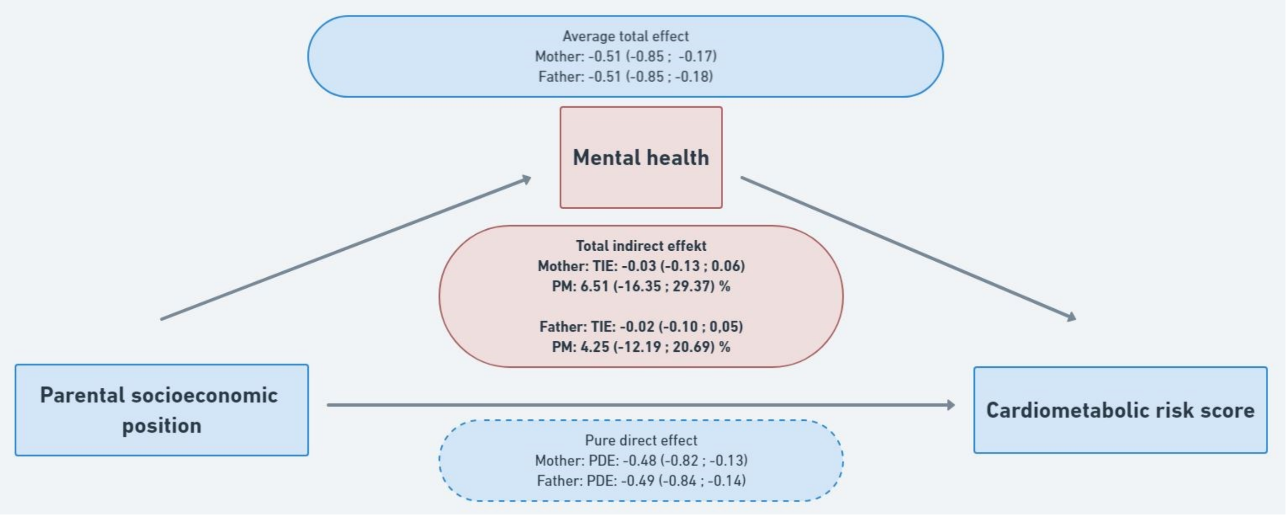


TIE, total indirect effect. PM, proportion mediated. PDE, pure direct effect. All estimates are presented with 95 % confidence intervals.
